# Mammary Paget's Disease Mimicking Benign and Malignant Dermatological Conditions: Clinical Challenges and Diagnostic Considerations

**DOI:** 10.7759/cureus.65378

**Published:** 2024-07-25

**Authors:** Renee Scott-Emuakpor, Setareh Reza-Soltani, Sana Altaf, Kaushik NR, Faustyna Kołodziej, Susana Sil-Zavaleta, Monica Nalla, Muhammad Naqib Ullah, Maha R Qureshi, Yasmin Ahmadi, Ali Rezvani, Humza F Siddiqui

**Affiliations:** 1 Dermatology, University of Miami Miller School of Medicine, Miami, USA; 2 Advanced Diagnostic and Interventional Radiology Center (ADIR), Tehran University of Medical Sciences, Tehran, IRN; 3 Internal Medicine, Deccan College of Medical Sciences, Hyderabad, IND; 4 General Medicine, Rajiv Gandhi Government General Hospital, Chennai, IND; 5 Medicine, Pomeranian Medical University in Szczecin, Szczecin, POL; 6 Dermatology, Universidad Anahuac, México City, MEX; 7 Dermatology, Hospital Ángeles del Pedregal, México City, MEX; 8 Surgery, Rajiv Gandhi University of Health Sciences, Bengaluru, IND; 9 Internal Medicine, Mayo Hospital, Lahore, PAK; 10 Medicine, National Health Service, Birmingham, GBR; 11 Medicine, Royal College of Surgeons in Ireland - Medical University of Bahrain, Muharraq, BHR; 12 Anesthesiology, Case Western Reserve University School of Medicine, Cleveland, USA; 13 Internal Medicine, Jinnah Sindh Medical University, Karachi, PAK

**Keywords:** mammary paget's disease (mpd), paget's disease of the breast, eczema, ductal carcinoma, paget cells

## Abstract

Mammary Paget’s disease (MPD) or Paget’s disease of the breast is a rare dermatological malignancy of the nipple-areolar complex that manifests with a spectrum of symptoms spanning from itching and redness to more severe indications such as breast lump, nipple-areolar complex destruction, or nipple discharge. It is predominantly associated with an underlying ductal carcinoma in situ or invasive ductal carcinoma. MPD often masquerades as other benign and malignant dermatological conditions, including eczema, atopic dermatitis, psoriasis, and squamous and basal cell carcinomas, leading to delayed diagnosis and inappropriate treatment. Only one-third of the patients present with a palpable lump; therefore, advanced age with chronic and unilateral lesions should raise concern for MPD. Our review article presents case reports of MPD imitating other skin conditions and underscores the key findings of clinical features and diagnostic workup to help differentiate the condition. A literature review revealed that studies emphasize caution regarding the sole use of mammography and ultrasound in diagnosing MPD, particularly in cases lacking a palpable lump. This highlights the MRI as a superior and more accurate imaging tool. However, any suspicious lesion must be biopsied to allow histopathological and immunohistochemical examination, since there are some cases where MRI findings were negative in the presence of a biopsy-proven MPD. This highlights the need for clinicians to investigate any suspicious lesion of the nipple or breast using the complete triple assessment approach to exclude an underlying malignancy. It is imperative to establish therapeutic guidelines to approach any nipple lesion to minimize the risk of misdiagnosing any underlying cancer, which can be potentially fatal if left alone.

## Introduction and background

Mammary Paget's disease (MPD), or Paget's disease of the breast, is a relatively rare condition described by Sir James Paget in 1874 as an eczematous ulcerative lesion with a clear yellowish exudate of the nipple frequently associated with underlying breast carcinoma [1,2]. He identified 15 female patients with similar chronic nipple lesions who had developed breast cancer. Initially, these changes were considered benign in nature, but subsequently, they were discovered to have malignant characteristics. A similar disease, known as extramammary Paget's disease, was observed in the external genitalia of both men and women. Despite sharing histological characteristics, their underlying etiologies are different [2,3].

**Figure 1:**
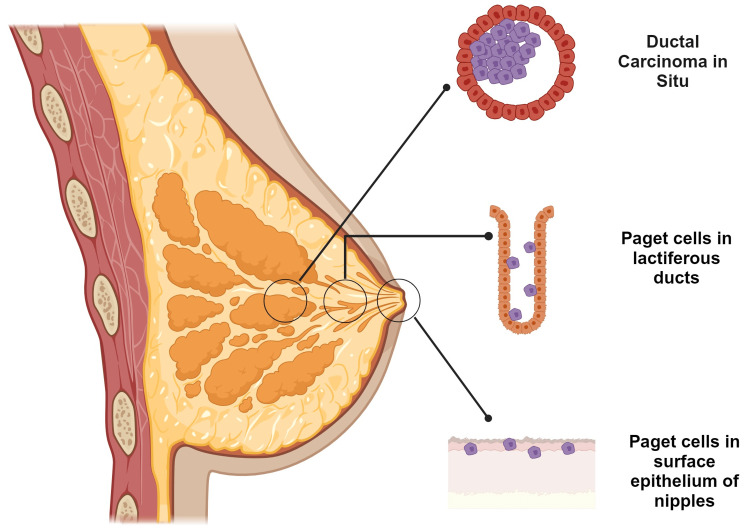
MPD with underlying carcinoma MPD: mammary Paget’s disease Figure made using biorender.com Image Credit: Humza F. Siddiqui

MPD is recognized as an atypical dermatological intraepithelial malignancy featuring large epithelial adenocarcinoma cells, termed Paget cells, located within the squamous epithelium of the nipple, which can spread into the areola and neighboring skin [4]. Paget's disease can also manifest in accessory nipples and ectopic breasts [5]. This uncommon condition affects predominantly postmenopausal females in their fifth or sixth decade of life. The frequency of this disease comprises between 1% and 3% of total breast cancers. More than 90% of cases are associated with underlying ductal carcinoma in situ (DCIS) or invasive ductal carcinoma (IDC), conventionally located centrally or multifocally. A prognosis is established based on the presence or absence of underlying invasive cancerous tissue. Bilateral synchronous tumors occur in about 1% of all breast cancers, which limits the data about the course of the disease. Only 1% of the documented cases of MPD are diagnosed in men with an average age of 68 years. Interestingly, some epidemiological data suggests that the incidence of Paget's disease has decreased by 45% in the last two decades, which is attributable to the early detection of DCIS due to mammography screening [6,7]. The literature identifies three categories of Paget's disease based on the presence and scope of associated disease: (1) Paget's disease correlated with underlying DCIS in the lactiferous ducts within 2 cm of the nipple, (2) MPD associated with invasive carcinoma located in the breast extending ≥2 cm from the nipple-areolar complex, and (3) MPD without underlying carcinoma (Figure 1) [8].

It is essential to be aware of the multifocality of this disease and mimicking breast carcinomas. Commonly, this condition is misdiagnosed and overlooked, as it may deeply resemble many typical skin rashes. The diagnostic process is mainly focused on clinical presentation and physical examination. However, it is essential to prioritize a medical history that centers around the duration of symptoms. Extended diagnosis of any persistent unilateral nipple abnormalities might be performed through methods such as cytological scraping of the nipple, MRI, or biopsy that allow histopathological examination and immunohistochemical staining. Although radiological tools have improved, each suspicious lesion of the breast must be biopsied to avoid the progression of malignancy with timely diagnosis [9,10]. Ashikari et al. identified that the average time period for diagnosis and treatment of MPD was six to 11 months, as compared to only one to two months for typical ductal carcinoma [11]. In this article, we aim to review the diagnostic parameters of MPD and highlight benign and malignant skin conditions that imitate Paget's disease for the consideration of clinicians and pathologists in making a timely diagnosis.

## Review

Pathogenesis

The pathogenesis of MPD is still a matter of debate, and numerous theories have been proposed to demonstrate the pathogenesis of Paget's disease. However, two key theories that explain the potential development of Paget’s disease have garnered wide acceptability [12,13].

Epidermotropic theory proposes that Paget cells arise from underlying mammary adenocarcinoma and that neoplastic ductal epithelial cells migrate through the ductal system of the breast to reach the epidermis of the nipple. Many studies have depicted that ductal epithelial cells and Paget cells have similar immunohistochemical staining properties, although epidermal keratinocytes of the surrounding nipple tissue stained differently than Paget cells. Furthermore, some early histological studies have exhibited ducts containing carcinoma cells, directly linked with nipple-expressing Paget cells. Overexpression of the human epidermal growth factor receptor (HER2/neu) on the surface of Paget cells occurs in a large proportion of PBD cases. Pelorca et al. studied 85 patients with MPD and found the HER2 or luminal HER2 molecular subtype in 83.5% of cases. It is hypothesized that epidermal keratinocytes secrete a motility factor called heregulin-α, which binds to the HER2 receptor and guides malignant cells to the surface. This hypothesis is supported by the high prevalence of underlying breast carcinoma in over 90% of MPD cases [14-17].

**Figure 2:**
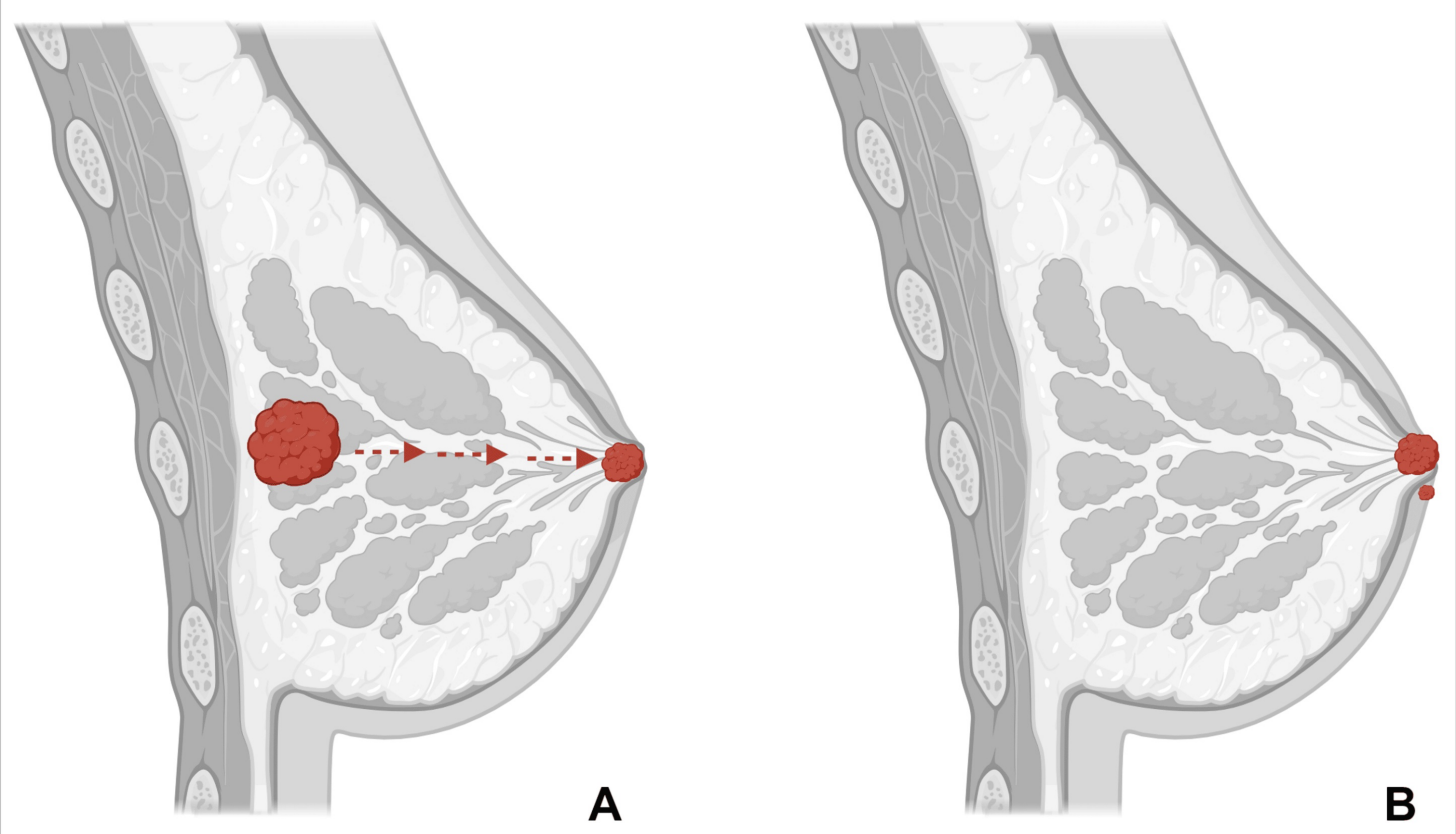
Pathogenesis of MPD (A) Epidermotropic theory: Paget cells originate in the underlying adenocarcinoma, and neoplastic epithelial cells migrate through the ductal system of the breast to reach the nipple epidermis. (B) Intraepidermal transformation theory: Paget cells originate through degeneration or in situ transformation of epidermal keratinocytes in the nipple-areola complex. MPD: mammary Paget’s disease Image made using biorender.com Image Credit: Humza F. Siddiqui

Conversely, the less favored theory is the intraepidermal transformation theory. According to this theory, epidermal keratinocyte carcinogenesis can develop independently of any underlying breast malignancy through the degeneration of existing cells or in situ transformation. This thesis states that Paget cells are a malignant form of pluripotent keratinocyte stem cells or apocrine gland ductus cells. Few studies have depicted Paget lesions with the absence of dermal invasion or the presence of desmosomes between Paget cells and adjacent cells, which prevent migration. In 1881, George Thinn created transformation theory, which also explained the progression of carcinogenesis without underlying carcinoma. He claimed that secretions from the breast ducts continuously damage the epithelium, which causes the transformation of keratinocytes into malignant cells. One of the researchers demonstrated specific cells called pre-Paget cells that appear intermediate between keratinocytes and Paget cells. It might be an affirmation of his thesis, suggesting that the epidermal cells can acquire the characteristics of ductal cells as they undergo the malignant transformation. As of now, despite extensive research, direct evidence validating either theory has not emerged, and epidermotropic theory has garnered the most acknowledgment (Figure 2) [2,3,15,18-20].

Risk factors

MPD shares the same risk factors as other breast carcinomas, including advanced age, obesity, alcohol consumption, familial genetic mutations including BRCA1 and BRCA2, prior chest radiation exposure, hormone replacement therapy, prolonged oral contraceptive use, and family history of breast cancer. A multi-center retrospective study by Zheng et al. focused on Chinese women with primary breast cancer and examined the demographic and risk factor differences between MPD and other types of breast cancer. The study compared several factors, including age, menstrual status, education level, parity, breastfeeding history, age at menopause, family history of breast cancer, and basic metabolic rate. The findings revealed that these demographic factors and risk exposures were largely similar in both groups [21]. However, Jamali et al. reported that the peak incidence of MPD occurs five to 10 years later than the peak incidence of invasive breast carcinoma [22]. Additionally, more patients with MPD have been reported to be nulliparous compared to other women with breast cancer [23].

Diagnosis

Clinical Presentation

MPD usually presents as dermatological changes in the nipple area complex (NAC), including pruritus, eczema, and erythema. It is a well-demarcated lesion that gradually evolves centrifugally into an erythematous or eczematoid plaque. Unlike non-cancerous eczema, which is bilateral, Paget’s disease is almost always unilateral [8,11]. As the disease progresses, it can involve skin erosion, ulceration, bloody or serous discharge, and flattening or inversion of the nipple. Crusted lesions, scaling, fissuring, and skin dimpling have been described in advanced cases. The rash can eventually expand up to 15 cm in diameter [1,24]. Physical symptoms are often preceded by unusual pain or a burning sensation in 15% to 20% of the cases [25,26]. The lesions often manifest in the nipple and spread to involve the adjacent areolar complex. However, few cases of extension of the lesion into the perimammary skin and opposite breast have been presented [27,28]. Hyperpigmenting lesions resembling superficial melanoma have been reported [29]. A dermoscopic examination of MPD lesions revealed that pink, unorganized patches and white lines were major predictors of the disease. Pigmented lesions depicted gray granules and dots, while ulcers and white scales were present among non-pigmented lesions [30].

Due to the possibility of a normal-appearing nipple with itching and an eczematous rash in Paget’s disease, this cancer may get misdiagnosed as a dermatological condition like dermatitis or eczema. Often, in such cases, women are managed with topical regimens while the actual disease remains undiagnosed [8,31]. Ideally, persistent itching, pain, and erythema of the nipple without any underlying cause should raise the suspicion of MPD, requiring the need for further evaluation [32].

A clinical study of 52 women with histologically proven Paget’s disease revealed that the majority of them had a nipple mass on examination, followed by redness, itching, and ulceration of the nipple, and the least common finding was bleeding and discharge from the nipple [33]. Apart from these findings on examination, axillary lymph node involvement can also be seen in advanced cases. A study of 20 patients with MPD indicated that half of the patients had axillary lymph node metastasis in association with a breast lump, while 12.5% of patients had axillary lymph node involvement without any lump [23,34]. A study reported that 113 patients presented with palpable masses with and without eczematoid nipple changes, and about 13% of patients with no palpable masses had axillary lymph node metastases [11]. Similarly, Fu et al. reported that 96% of patients with no palpable mass were diagnosed with Paget's disease with underlying carcinoma [35].

Imaging

If skin changes do not improve with corticosteroid treatment within two weeks, further diagnostic imaging and biopsy are recommended [2]. An effective workup includes high-quality imaging to rule out malignancy due to the strong association between MPD and breast carcinoma [8]. Research has shown that patients with Paget’s disease who have a palpable mass tend to have multifocal disease, while those without a mass may still have multifocal or multicentric disease [36]. Mammography is the preferred initial imaging modality for detecting potential cancer in MPD cases, with a breast ultrasound being considered if the mammogram results are inconclusive [2]. Patients who do not show any abnormalities on mammograms or ultrasounds and do not have any palpable masses are advised to undergo breast MRI [37].

Mammography is crucial for diagnosing and managing Paget's disease, but it may not always detect abnormalities and can appear normal in up to 50% of patients [8]. The common mammographic findings associated with MPD include thickened skin around the nipple-areolar area, differences in density, retracted nipples, a distinct lump, and/or microcalcifications [2,37]. In the study by Pelorca et al., mammography was performed in the majority of patients (85.9%), revealing abnormalities in 87.7% of cases, with microcalcifications being the most common finding (58.9%), followed by nodules (37%) [38]. Challa and Deshmane studied 20 women with MPD. Mammography was conducted on 11 patients, with two individuals showing no abnormalities. Among the nine patients with identified mammographic abnormalities, five exhibited fine microcalcifications, two had underlying masses, and two displayed increased skin thickening around the nipple. Two patients demonstrated multicentric calcifications involving more than two quadrants. All in all, multicentricity was observed in five cases (25%) [34]. Moreover, some studies report mammographically hidden malignancies at rates ranging from 15% to 65% [29,32,39]. Beyond identifying underlying masses, or DCIS, mammography is valuable for monitoring patients who have undergone conservative breast surgery to exclude recurrence [37]. Mammography has a sensitivity of 97% in detecting underlying cancer in Paget’s disease, where there is a clinically appreciable breast lump. However, in the absence of a lump, it can detect an underlying malignancy in only half of the cases [2,4,40]. Therefore, a negative mammogram does not rule out the presence of tumors [1].

Ultrasound is utilized not only to validate the mammography results in cases of Paget's disease but also in instances where the mammogram shows no abnormalities. Ultrasound findings may include parenchymal heterogeneity, hypoechoic areas, dilated ducts, distinct mass, and cutaneous thickening [37,40]. Ultrasound can also be used to evaluate the appearance of the axillary nodes [2]. Microcalcifications are often not detectable on ultrasound. Occasionally, a region of DCIS with pleomorphic calcifications seen on mammography alongside an underlying mass may be more clearly visualized on ultrasound. However, in certain situations, despite findings on mammograms, the ultrasound results may be inconclusive and might only indicate skin thickening in that region [37]. Furthermore, combining ultrasound with mammography has not shown increased sensitivity in detecting underlying lesions compared to mammography alone. However, ultrasound can aid in further characterization or guide core biopsies of any palpable or mass-like abnormalities identified on mammography [3]. Günhan-Bilgen and Oktay studied 52 MPD cases, and ultrasound identified 43 masses in 35 individuals, which displayed irregular or lobulated shapes (95%) with no posterior shadowing [32].

MRI is extremely sensitive to detecting breast tumors, particularly when mammography and ultrasound are unremarkable. It can reveal papillary-areolar complex thickening and nipple enlargement and identify in situ ductal lesions and invasive tumors, even in clinically unsuspected cases [40,41]. There can be variations in patterns of enhancement, including irregular, asymmetric, or discoid. Contrast-enhanced breast MRI may be employed to determine the extent of disease in patients with positive ultrasound or mammogram findings, particularly when breast-conserving surgery (BCS) is considered. In cases of suspected or confirmed MPD, MRI can help identify hidden, multifocal (numerous foci of carcinoma in the same quadrant of the breast), or multicentric (more than one foci in the separate quadrant) lesions that may not be evident on clinical examination or imaging tests [2]. In the study of Pelorca et al. with 85 MPD cases, ultrasonography was performed in 79 patients (92.9%), detecting solid lumps as the primary abnormality in over 70% of cases. A subset of patients underwent breast MRI, where tumors were identified in almost 70% of cases and NAC thickening in around 43%. There were no significant differences between clinical and hidden or pathological forms of Paget’s disease regarding the frequency of imaging tests such as mammography, breast ultrasonography, and MRI [38].

A study by Siponem et al. involving 58 patients with Paget’s disease showed that MRI is the most sensitive modality for detecting infiltrating carcinoma (100%) and DCIS (44%), followed by mammography (74% and 39%, respectively) and ultrasound (74% and 19%) [2,42]. Morrogh et al. found that in 34 women, where 32 had biopsy-proven MPD, MRI failed to detect cancer in three patients [39]. On the other hand, due to its high sensitivity but relatively lower specificity, MRI may identify abnormalities that could lead to unnecessary mastectomy rather than breast-conserving therapy. Therefore, if MRI is considered, it should only be performed at a facility capable of conducting MRI-guided biopsies, and patients should be informed about the high false-positive rate of breast MRI and the potential need for further biopsies [3]. According to a case report of a 52-year-old woman whose initial presentation was simply a change in the color of nipple skin, the MRI was positive for underlying carcinoma in the setting of a negative mammogram and no clinically palpable lump. A biopsy of the nipple suggested MPD, while an MRI revealed an underlying, extensively spreading DCIS as diffuse segmental enhancements [43]. While Paget's disease is primarily diagnosed based on clinical presentation, a negative imaging finding may not definitively rule out occult breast cancer [37].

Histopathological and Immunohistochemical Features of MPD

Tissue scraping from the affected area is a reliable screening technique that helps in the rapid diagnosis of MPD due to the high nuclear-to-cytoplasmic ratio and vacuolated cytoplasm of the large cell. Biopsy techniques that can be used for diagnosis are superficial shave biopsy of the epidermis, wedge biopsy, and punch biopsy. A wedge biopsy is preferable as it includes adequate epidermis; a shave biopsy in ulcerated cases will show insufficient Paget cells; and a punch biopsy contains stroma and minimal epidermis for examination [44]. The standard method is surgical excision, especially in cases with unremarkable biopsy. A full-thickness biopsy is used in patients with nipple-areolar skin changes [45].

Paget cells are the hallmark of MPD. These cells are intraepithelial adenocarcinoma cells of variable sizes that are often located in the basal layer without any intracellular bridges connecting them to the neighboring cells. These cells are sporadically arranged as small clusters and large sheaths in nest-like arrangements or gland-like formations, often entirely substituting the epidermal cells. Paget cells are mucin-positive and contain neutral mucopolysaccharide cytoplasm. Microscopic features present as pale to clear abundant vacuolated cytoplasm with hyperchromatic pleomorphic nuclei and one to two prominent nucleoli [46]. Dense inflammatory infiltrates comprising lymphocytes are often evident in the superficial dermis. Toker cells are benign cytoplasm-rich epithelial cells that evolve through the sebaceous glands. Toker cells are observed in 10% of normal nipple tissue, imitating Paget cells, and are rarely also found in supernumerary nipples and apocrine glands [47].

Immunohistochemical staining is an effective tool that plays a substantial role in delineating molecular subtypes of MPD for the purposes of staging, opting for appropriate therapy, and estimating prognosis. It also aids in distinguishing MPD from other nipple-associated diseases. The majority of studies depict HER2 as widely overexpressed among MPD cases. Estrogen receptor (ER) and progesterone receptor (PR) positivity were found to be 40% and 30%, respectively, in a study. A study compared MPD with other breast cancers and revealed that Luminal A and B subtypes were more predominant in other breast cancers, while MPD stained positive for HER2 consistently. Ninety-five percent of Paget cells stained positively for cytokeratin. GATA-3 staining can also be utilized, as Paget cells tend to spread to the nipple epidermis by chemotactic movement through heregulin-α [48-54].

MPD mimicking dermatological conditions

**Figure 3:**
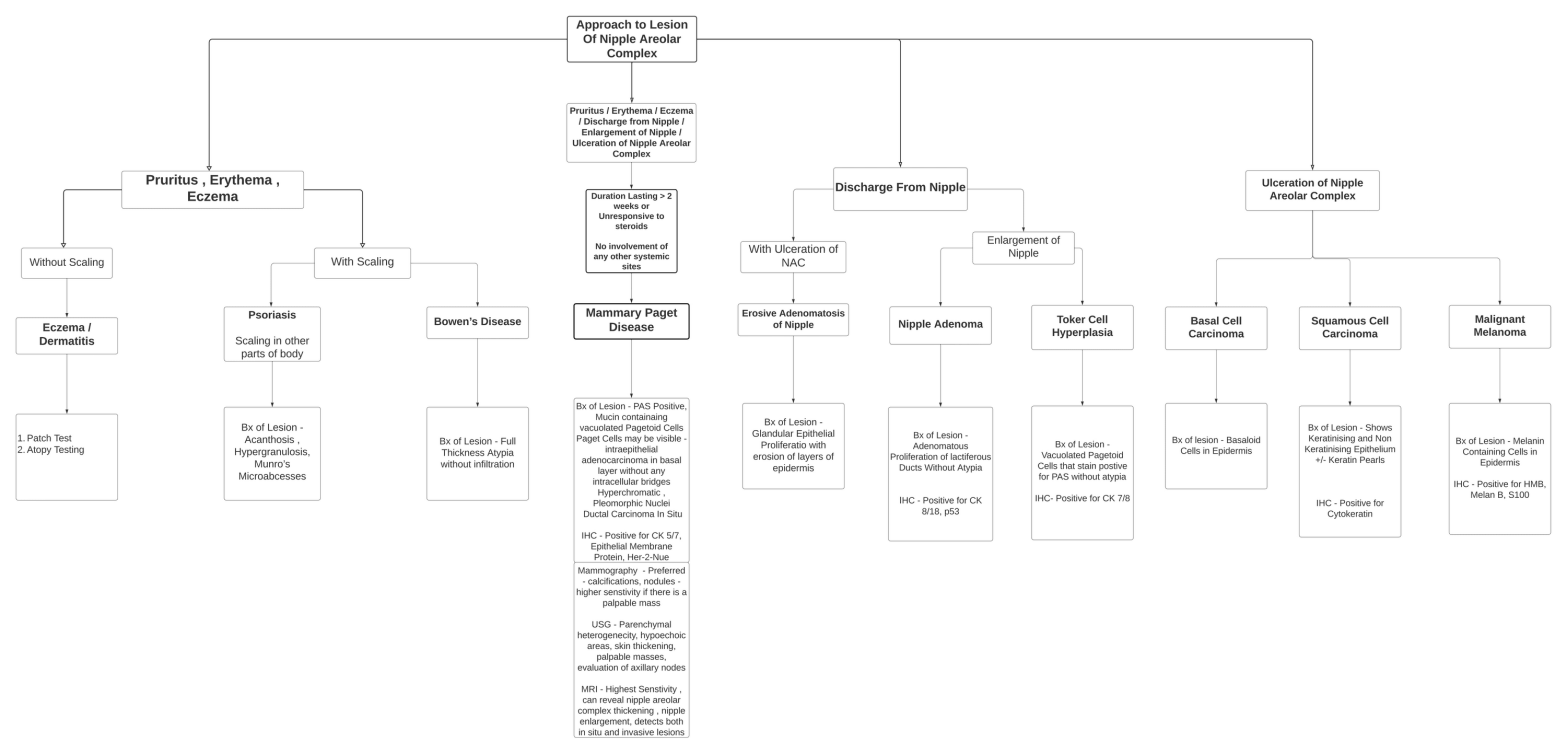
Diagnostic flowchart of the differential diagnosis of nipple-areola complex diseases Image Credit: Renee Scott, Sana Altaf, and Kaushik NR

MPD can resemble several benign and chronic skin conditions, including chronic eczema, atopic dermatitis, contact dermatitis, psoriasis, erosive adenomatosis, squamous cell carcinoma, basal cell carcinoma, and malignant melanoma (Figure 3, Table 1).

**Table 1:** Differential diagnosis of skin lesions of the nipple-areola complex PAS: periodic acid-Schiff; CK: cytokeratin; CEA: carcinoembryonic antigen; HER2/neu: human epidermal growth factor receptor 2; HMB: hydroxymethylbutyrate; HPE: histopathological examination; IHC: immunohistochemistry; MPD: mammary Paget’s disease; IgE: immunoglobulin E [56-100]

Disease	Clinical features	Diagnostics	
		HPE	IHC
MPD [44-54]	This disease may present with scaling, eczema, erythema, ulceration, erosion, hyperpigmentation of the nipple alveolar complex, and discharge preluding from the nipple.	In order to diagnose this disease an incisional biopsy that depicts ductal carcinoma must be performed. A biopsy will show PAS-positive mucin-containing vacuolated pagetoid cells.	Positive for CK 5/7, epithelial membrane protein, CEA, and HER2/neu overexpression.
Chronic eczema [55-61]	This disease may present with lichenification, erythema, and hyperpigmentation.	In order to diagnose this disease a patient's medical history must be analyzed as well as patients, symptom descriptions, familial history, triggers, and physical examinations.	Patch testing, skin biopsy, and blood tests indicate elevated levels of IgE antibodies.
Atopic dermatitis [56,57, 62-65]	This disease may present in the form of erythema, papules-vesicles, erosions, and pruritus.	In order to diagnose this disease a clinical examination must be performed as well as patient’s history should be taken into account. The following must be noted on the biopsy: irregular acanthosis, spongiosis, inflammatory cell infiltrate in the dermis, and elevated IgE levels.	-
Contact dermatitis [57,63-68]	This disease may present in the form of erythema, papules-vesicles, erosions, and pruritus.	In order to diagnose this disease clinicians must first identify the contacting agent and perform a patch test.	-
Psoriasis [69-73]	This disease may present with defined patches with erythema, scales, forms of infiltration, and pruritus.	In order to diagnose this disease a clinical examination must be performed, as well as a biopsy positive for regular acanthosis, hypogranulosis, Munro microabscesses, and Kojog´s pustules.	-
Bowen's disease [88-89]	This disease may present with patchy lesions, as well as slow growth, irregular borders, scaling, itching, and burning.	In order to diagnose this disease, a clinical examination must be performed as well as dermoscopy of irregular pigments, biopsies, and histopathological examination of biopsies.	-
Erosive adenomatosis of the nipple [87]	This disease often presents with ulcers on the surface of the nipple or areola, discharge pain, and itching.	In order to diagnose this disease a clinical examination as well as an analysis of the patient’s history, imaging studies, specifically mammography and breast ultrasounds. Lastly, a histopathological examination of a biopsy must be performed.	-
Nipple adenoma [53,54,77-84]	This disease often presents with firm nodules, crusting, or erosion of the nipple. Furthermore, the image seen in a mammography may depict sub-alveolar calcifications.	The diagnosis is determined via biopsy. The adenomatous proliferation of lactiferous ducts with surrounding myoepithelial cells without cellular atypia. There will be no PAS staining found on vacuolated cells.	Positive for CK 8/18 and p53.
Toker cell hyperplasia [74-76]	This disease often presents with enlargement or thickening of the nipple, papillomatous growth, color changes, itching, burning, nipple discharge, and hyperplastic changes in the ductal epithelium of the nipple.	The diagnosis is determined via biopsy. HPE: vacuolated pagetoid-like cells must stain positive for mucin with PAS without any cellular atypia. The stain may show normal or hyperplastic cells.	Positive for CK 7/8 and epithelial cell membrane antigen. Negative for p53 and HER2.
Malignant melanoma [51,53,54,97-100]	This disease often presents as a black ulcerative or crusting erosion of the nipple-areolar complex.	A biopsy is needed in order to differentiate pigmented varieties of MPD. HPE: melanin-containing cells in the epidermal layers with or without invasion. Stains that are negative for specific stains such as mucicarmine.	Positive for HMB, Melan B, S-100.
Invasive squamous cell carcinoma of the nipple [90,91]	This disease may present as scaly ulcerative lesions of the nipple alveolar complex.	A biopsy of the affected breast is needed for a definitive diagnosis. HPE - shows keratinizing and non-keratinizing epithelium in the absence of reactive epithelialization.	Positive for cytokeratin.
Basal cell carcinoma [92-95]	This disease may present as an ulcerative lesion of the nipple alveolar complex. This disease may also present with or without excoriation.	Pigmented basaloid cells in the epidermis extend to the dermis.	Positive for Ber-EP4.

Benign Conditions

Eczema: One of the main clinical presentations of MPD is eczema [55]. Several case reports have been published highlighting the cases of MPD misdiagnosed as eczematoid dermatitis, delaying appropriate treatment. Chronic eczema is usually bilateral and diagnosed with an extensive patient history and physical examination. Skin patch testing to identify allergens and biopsy compounded with elevated levels of serum IgE antibodies are usually used to make a definitive diagnosis [56-58].

Bansal et al. reported a case of a 46-year-old woman who presented with an extensive erythematous plaque covering her entire left breast and sub-mammary region, gradually progressing over time. The surface of the lesion was covered by ulcers and hemorrhagic crusts, obliterating the entire NAC. The patient was prescribed multiple steroids and antibiotic ointment over the course of three years, owing to repeated misdiagnosis, until finally the diagnosis of MPD was reached with a biopsy [59]. Kanwar et al. described a similar, long-standing case in which the patient was persistently incorrectly diagnosed for 14 years [60]. A 24-year-old young woman presented with progressive ulcerated eczema that completely distorted the nipple and areolar region and was prescribed a topical steroid a year ago by the local physician [61].

Atopic dermatitis: The frequency of involvement of the nipple in atopic dermatitis can be up to 23% [57]. In acute phases, this subtype of eczema can manifest clinically as bilateral dermatoses, constituted by erythema, papules, vesicles, and pruritus, with or without previous typical outbreaks of atopy, with a predominance in adolescent women [62,63]. In chronic atopy, the skin will develop lichenification due to continuous scratching [57,64]. The biopsy to rule out Paget´s disease must be obtained from full-depth tissue from the areola and the nipple [2,8]. Punch biopsies may be used, although false negative results might be obtained [56,65]. The histology in atopic dermatitis, as in eczema, is defined by acanthosis, spongiosis, and lymphocytic exocytosis; in the dermis, we may encounter perivascular lymphocytic and eosinophilic infiltration [63]. In chronic dermatoses, we may find hyperkeratosis and more mast, Langerhans cells, and eosinophilic infiltrates [57]. Immunochemistry findings include TH-2 cytokines related to atopic dermatitis, such as IL2 and IL13, expressed in the epidermis and in the perivascular dermis [63].

Allergic and irritative contact dermatitis: Nipple eczema is proposed by some authors as a major sign of atopic dermatitis, but allergic contact dermatitis must be ruled out if the nipple eczema is continually manifesting after the standard treatment [63,66]. First, we must look for the contacting agent. Patch tests have been reported to be positive for ion metals or components on fabric cleansers and nipple creams [6466]. We may consider that clinical manifestations of skin allergy such as vesicles, erythema, and scratching tend to appear 24-48 hours after the exposure [57,64]. As skin friction is a risk factor for developing contact dermatitis, in irritative eczema, occupational hazards and habits such as breastfeeding, lactation pump use, or exercise must be inquired about [64,67,68]. The microscopic changes in contact dermatitis in the epidermis are eosinophilic spongiosis, acanthosis, and lymphocytic exocytosis, with lymphocytic and neutrophil perivascular infiltration [57,65].

Psoriasis: In this chronic, autoimmune, inflammatory disease, the most common clinical presentation corresponds to plaque psoriasis. It manifests as well-defined patches with different grades of erythema, scale, infiltration, and pruritus, mostly on the extensor surfaces [57,69]. Psoriasis is related to the Koebner phenomenon, an isomorphic reaction characterized by the appearance of plaques induced by different physical stimuli. It may affect the nipple and areolar complex in rare cases [70]. A higher incidence of psoriasis in patients with previous breast cancer has been previously published [71]. The histopathological changes in psoriasis correspond to regular acanthosis, diminished stratum granulosum, parakeratosis, subcorneal neutrophil infiltration (Kogoj´s spongiform pustules), and neutrophil infiltration in the stratum corneum (Munro's microabscesses) [72]. Interestingly, an uncommon case of concurrent occurrence of psoriasis and MPD was reported in an elderly female patient [73].

Toker cell hyperplasia: Di Tommaso et al. aimed to investigate the prevalence of atypical Toker cells and determine the physical markers that strictly differentiate them from malignant cells deriving from Paget's disease. Toker cell hyperplasia usually presents as a small, isolated lesion that is non-pigmented, non-irritating, and firm. Furthermore, this condition may be observed in one or both nipples and is more commonly seen in women. In the study, over 390 patients underwent a breast mastectomy, and Toker cells were found in the nipples of about 40 cases. These Toker cells were further divided into three categories: normal Toker cells making up 60%, hyperplastic Toker cells accounting for 27.5%, and hyperplastic and atypical Toker cells found in 12.5% of all cases. When analyzed via immunohistochemical techniques, it was revealed that the Toker cells were positive for ER and PR but negative for CD138 and p53. Interestingly, some atypical Toker cells possessed slight HER2/neu immunoreactivity. Alternatively, the opposite staining pattern was noted when compared to Paget cells. Furthermore, both Toker and Paget cells tested positive for cytokeratin 7 (CK 7) and epithelial membrane antigen but negative for p63. Hence, in order to distinguish atypical Toker cells from Paget cells, researchers used a combination of CD138 and p53 staining, which proved effective. The study underscores the crucial role of accurate diagnosis in understanding the relevant characteristics of Toker cells and how they differ from their malignant counterparts in Paget's disease [74].

Ramos et al. presented a case of an 83-year-old woman who had been referred to gynecological services for an eczematous lesion of the left nipple for the past 17 years. It was nonpruritic, erythematous, and non-tender. Critical differential diagnoses were chronic eczematous conditions of the nipple and Paget's disease of the nipple. A punch biopsy was conducted to obtain a definitive diagnosis, showing epithelioid cells isolated from the epidermis staining negative for PAS stain. Immunohistostaining for CK 5/7 turned out to be positive, but not for p53. Hence, a conclusive diagnosis of Toker cell hyperplasia was made instead of Paget's disease of the nipple [75]. Similarly, the case reported by van der Putte et al. characterized a 47-year-old female who presented with an eczematous lesion in the right areola for the past 10 years, which, on biopsy, showed a monomorphic collection of cells in the epidermis, leaving other layers and surrounding tissue unaffected. Initially, though described as a variant of MPD, it was subsequently interpreted as a hyperplasia of mammary glands in the epithelium. The laboratory results in the diagnosis strongly suggest Toker cell hyperplasia [76].

Nipple adenoma: MPD and nipple adenoma, though rare, can present with strikingly similar features that can be differentiated only through histological and immunohistochemical studies [77]. Nipple adenoma is a rare benign condition of the breast associated with lactiferous duct proliferation that can present as a firm nodule, crusting erosion or ulceration, and nipple discharge [78]. It can present with or without a palpable mass below the nipple [79]. Nipple adenoma, when associated with marked proliferation of myoepithelial cells, can result in local inflation and enlargement but no metastasis, leading to destruction of the nipple resulting in erosion, mimicking Paget’s disease clinically [80]. The clinical differences between nipple adenoma and MPD are limited; hence, it is a crucial diagnostic dilemma. A few differences highlighted in the literature are that Paget’s disease is quicker in progression with severe excoriation and itching, while nipple adenoma has a mass more confined to the nipple relative to Paget’s disease and has a clearer exudation [77,81].

Due to the nonspecificity of clinical features, the burden of differentiating between a nipple adenoma and MPD lies on the histopathological features. A nipple biopsy confirmation and subsequent surgical excision remain the gold standard for nipple adenoma [82]. Nipple adenoma is often occult in mammography; however, it occasionally presents with calcifications [83]. On biopsy, nipple adenoma macroscopically is found in the retroalveolar region. It is identified as a gray, non-encapsulated lesion with ductal and adenomatous proliferation surrounded by epithelial and myoepithelial cells. Immunohistochemically, nipple adenoma stains positive for CK 8/18, CK 5/6, and p63 [84]. On the other hand, the hallmark of MPD is the presence of Paget cells, which are malignant epithelial adenocarcinoma cells of variable size. They are ovoid and vacuolated and contain mucin; hence, stains are positive for periodic acid-Schiff (PAS) stain. On subjecting the tissue biopsy to immunohistochemistry (IHC), it mainly stains positive for CK 7/20 and may stain positive for carcinoembryonic antigen (CEA) and cytokeratin [53,54].

Ono et al. described a case of giant nipple adenoma in a 63-year-old female who presented with erosions and excoriations that were initially presumed to be a case of MPD. However, later, the case was diagnosed as a nipple adenoma based on histopathological examination showing proliferation of myoepithelial cells staining positive for CK 5 and 14 [85]. Rodriguez et al. described a case of a 35-year-old woman with no medical co-morbidities who presented with a palpable mass, pruritus, and excoriation of the nipple. Mammography revealed a sub-areolar mass. After six months, there was an increase in size, prompting the need for an excision biopsy, with Paget’s disease and nipple adenoma being key differentials. A histopathological examination later revealed it to be a case of benign intra-ductal papilloma [78]. Abbas et al. discussed a case of a 36-year-old woman who presented with left nipple erosion. On examination, extensive erosion of the left nipple without any palpable mass was identified. Clinical differentials included MPD and skin malignancy. On an excision biopsy, it was found to be a benign nipple adenoma. These cases reflect that although nipple adenoma is a benign diagnosis, its nonspecific clinical presentation and features resembling Paget’s disease warrant the need for biopsy and resection [86].

Erosive adenomatosis of the nipple: Gnangnon et al., in a case report, illustrated the disease's rarity and essential characteristics. Erosive adenomatosis of the nipple is a benign breast neoplasm characterized by nodules surrounding the nipple ducts. These nodules erode the nipple tissue, causing severe pain, milky discharge, and changes in the appearance of the breast, specifically the nipple region. The case report presents a 45-year-old woman admitted to the hospital with complaints of a growing mass on her left nipple. The patient is later diagnosed with erosive adenomatosis of the nipple and undergoes a nipple resection accompanied by breast reconstruction. Erosive adenomatosis of the nipple should be effectively treated and comprehensively analyzed alongside malignant nipple tumors such as MPD in order to draw up a definitive treatment plan. Erosive adenomatosis slightly mimics the clinical manifestations of malignant conditions of the skin and breast, including MPD. Surgery is the most effective treatment for this specific subtype of adenomatosis, with a favorable prognosis for the patient [87].

Malignant Conditions

Bowen's disease: Squamous cell carcinoma in situ, often referred to as Bowen's disease, is a non-invasive skin cancer that primarily impacts the epidermis. Bowen's disease shares similar characteristics with other skin conditions, such as psoriasis and eczema. In addition, Bowen's disease's clinical manifestations may often lead to a differential diagnosis of seborrheic keratosis, actinic keratosis, and a host of other benign skin conditions. Due to its similar phenotypic features and clinical presentation, Paget's disease is frequently referenced as a differential diagnosis of Bowen's disease. The physical manifestation of Bowen’s disease often possesses a scaley, rough, or crusted surface. The borders surrounding these asymmetrical lesions are often characterized as ill-defined, a distinguishing feature of the condition. In addition, the color of the lesion may vary from light pink to rugged brown or even a scarlet crimson on highly melanated individuals, such as those of African descent. However, it is essential to note that Bowen's disease may manifest differently from person to person, and the previously listed characteristics are not exclusive to making a diagnosis [88].

Barrutia et al. presented two unique cases that delve into a rare subtype of Bowen's disease known as pagetoid Bowen's disease. This condition is exceptionally uncommon, accounting for less than 5% of all Bowen's cases. With very few cases documented in the current literature, this case report sheds light on the intricacy of this condition manifesting within this region of the breast, one of which is the first reported case of pagetoid Bowen's disease affecting the nipple area. The histopathological study of Bowen disease exhibits differentiating features, including full-thickness atypia of the epidermis and intercellular bridges. An immunohistochemical panel incorporating CEA, PAS, ER, PR, p63, gross cystic fluid protein, and epithelial membrane antigen needs to be performed to differentiate Paget's disease from pagetoid Bowen's disease. This indicates that a comprehensive analysis constituting an excisional biopsy of tumors and immunohistochemical staining are essential for accurate diagnosis in highly complex cases [89].

Squamous cell carcinoma: Primary squamous cell carcinoma of the nipple is rare and usually presents as a scaly erythematous lesion. It is similar to Paget’s disease of the nipple and requires careful consideration for a histopathological and definitive diagnosis. While nipple adenoma and malignant melanoma are clinical mimickers of Paget’s disease, squamous cell carcinoma has been reported to be a cytological mimicker of Paget’s disease [90]. Vohra et al. described a case of MPD that was initially diagnosed as squamous cell carcinoma on cytology. Histological and cytological examination of the excised specimen showed a mixed population of both keratinizing and non-keratinizing epithelial cells that led to the diagnosis of squamous cell carcinoma of the breast. On further examination, reactive changes of the squamous epithelium caused by Paget’s disease were recognized and confirmed immuno-histologically [91]. Sofos et al. described a case of a 36-year-old woman who presented with a scaly erythematous lesion of the right nipple-alveolar complex, which was investigated with MPD as the primary differential diagnosis. The lesion was excised and subjected to histopathological examination, where non-keratinizing epithelial cells were identified. However, it was later definitely diagnosed as squamous cell carcinoma of nipple-alveolar complex IHC [90].

Basal cell carcinoma: Basal cell carcinoma of the nipple-areolar complex, though a very rare entity, has been seeing a rising trend in incidence [92]. It is so rare that by 2010, only 34 cases of basal cell carcinoma of the nipple-areolar complex had been reported in the literature. It is usually a diagnosis of exclusion or an incidental diagnosis. It is a true masquerader of breast malignancy [93]. Basal cell carcinoma can present with excoriating lesions of the nipple-areolar complex with erosion mimicking MPD. When a suspicious lesion is subjected to biopsy, basal cell carcinoma shows layers of pigmented basaloid cells in the epidermis and dermis. It may also be associated with the deposition of melanin in the superficial layers of the skin [92]. In a case report by Sharma et al., a 48-year-old woman presented with a history of pigmented excoriating lesions of the right nipple-areolar complex for the past three months. The primary differentials were MPD and congenital melanocytic nevi. An excision biopsy was performed, and it showed basaloid cells in the epidermis and dermis with the deposition of melanin. An IHC with human melanoma black (HMB) 45 and CK 5/6 was performed, which turned out to be negative. Unexpectedly, a diagnosis of basal cell carcinoma was made and proceeded accordingly, reflecting the masquerading capacity of basal cell carcinoma [94].

Moennich et al. reported a case of a 47-year-old woman who had complained of spontaneous bloody discharge from her right nipple. On a shave biopsy, atypical basaloid cells were found and stained positive for antihuman epithelial antigen (Ber-EP4). Surgery proceeded with the histological diagnosis of basal cell carcinoma of the nipple-areolar complex. During the surgery, when the mass was subjected to a frozen section, the larger specimen showed features of atypical and pleomorphic ductal cells similar to Paget’s disease, and the larger specimen when subjected to IHC stained positive for CK 7 and HER2/neu. The initial plan was abandoned, and surgery for lumpectomy with nipple removal and adjuvant radiation was proceeded with [95].

Malignant melanoma: Pigmented MPD, a rare form of Paget’s disease, can clinically and histologically imitate malignant melanoma. Paget’s disease of the nipple, resulting in melanocytic stimulant release by pagetoid cells, can result in melanin deposition, impersonating a case of melanoma in situ. The deposited melanin pigment can be derived from the nearby epithelial cells. Melanoma initially presents as a black mole in the nipple that later spreads and ulcerates [96]. Histologically, MPD can resemble malignant melanoma if the cells have melanin incorporated into them. Special stains like mucicarmine can highlight the mucin-containing vacuoles in Paget cells but not those of malignant melanoma [51,54]. A definitive diagnosis can be derived from immunohistochemical staining using S100 and HMB for melanoma and CK 7 for Paget’s disease [96]. Malignant melanoma will not stain positive for ER and PR, unlike MPD [51,53,54]. The difficulty with differentiating Paget’s disease from melanoma lies not only in the similarities in the histopathology but also in the method of collection. Though MPD can be easily diagnosed using a core needle biopsy, malignant melanoma has a chance of dissemination on a fine or core needle biopsy. Hence, an excision biopsy is preferred in a suspicious lesion [97].

In the study conducted by Dehner et al., 10 cases of primary melanoma of the nipple were described. One of those cases was reported to have a coexisting MPD. Primary malignant melanoma of the nipple is so rare that pigmented MPD must be ruled out before considering melanoma [98]. Saito et al. reported a case of pigmented Paget’s disease that was initially misdiagnosed as malignant melanoma initially [99]. Similarly, Lee et al. reported a case of pigmented MPD that presented as a black mass over the nipple. It histologically showed neoplastic cells with pigmentation, leading to a diagnosis of malignant melanoma, resulting in a wide excision biopsy. When the excised lesion was sent for immunohistochemical analysis, it showed positivity for CK 7 rather than HMB and S100 as expected [100].

Management

The management of MPD varies with each patient’s clinical presentation and diagnostic findings. Treatment strategies for MPD are established by a multidisciplinary team, which normally consists of oncoplastic surgeons, radiologists, breast care nurses, medical geneticists, clinical psychologists, and palliative care experts. However, the mainstay intervention for MPD is surgery, whether it involves mastectomy or BCS, depending on many factors, such as the association with DCIS or IDC adjacent to the nipple-areolar region, clinical presentation, and quality of life [101,102]. The National Comprehensive Cancer Network (NCCN) guidelines are often used by physicians to guide the main treatments for MPD. The NCCN guidelines involve a management plan considering the presence of concurrent breast cancer, which ultimately determines the type of surgery suitable for the patient. The management of Paget's disease based on the NCCN guidelines involves subcategorizing patients based on their diagnostic investigations, such as through breast and NAC biopsies, depending upon their results. The treatment of DCIS involves either a BCS without lymph node surgery or a total mastectomy with sentinel lymph node biopsy (SLNB) instead of axillary dissection and +/- breast/nipple reconstruction. However, in cases where the breast cancer investigations are negative but solely positive for NAC Paget’s disease, a central lumpectomy including NAC with whole breast radiation therapy, a total mastectomy +/- SLNB with or without breast reconstruction, or a central lumpectomy including NAC +/- SLNB without radiation therapy is recommended [101,102].

Traditionally, mastectomy has been the treatment of choice; however, with recent advancements, BCS (wide local excision of the nipple-areola complex) with adjuvant therapy has emerged as an effective treatment modality among patients with no underlying carcinoma due to higher patient satisfaction rates. However, there is a significant concern about positive resection margins necessitating re-operation for another resection or complete mastectomy [103,104]. Kollmorgen et al. reported that 29% of MPD cases with no concurrent carcinoma presented with a peripherally located lesion that was not suitable for a wide local excision of NAC and had to undergo mastectomy [19]. BCS with wire-guided local excision is a viable surgical option among patients without a palpable mass and unremarkable mammography in combination with radiotherapy [25]. A study showed that the 10-year survival rates of patients undergoing BCS and mastectomy were roughly similar, 67% and 79%, respectively. However, the patient cohort for BCS (n=12) was significantly smaller as compared to mastectomy (n=102) [105]. The European Organization for Research and Treatment of Cancer Trial advocated that if a clear margin is achieved at excision, BCS with whole breast radiotherapy is a reasonable approach for MPD and localized DCIS with only a 5% recurrent rate at five years postoperatively [103]. Chen et al. established that a 15-year cancer-specific survival rate was 92% for mastectomy and 94% for BCS among patients with or without DCIS [106]. Dalberg et al. reported a 10-year disease-free survival of 85% for mastectomy and 94% for BCS [107]. Li et al. performed a meta-analysis including 685 patients and revealed a significant difference in the local recurrence rate between mastectomy and BCS, 5.6% and 13.2%, respectively. However, the authors suggested that a clear superiority of mastectomy over BCS cannot be delineated due to inconsistencies in diagnostic and adjuvant therapeutic modalities. The patients who had relapsed in this study progressed to invasive breast carcinoma, and invasive recurrence resulted in a poor prognosis among patients treated with BCS [108]. A SLNB is also always recommended when a patient is undergoing mastectomy to prevent dissection of the entire lymph node, provided an invasive component is exhibited at follow-up histopathological examination of the tissue [109].

Most studies highlighted the crucial role of radiotherapy in significantly contributing to the overall prognosis. In regards to MPD associated with DCIS, the 15-year overall survival and breast cancer-specific survival rate of MPD-DCIS patients who were treated with BCS and radiotherapy reached 90% and 97%, respectively [110]. A meta-analysis of 38 studies showed that BCS with radiotherapy significantly reduced the local recurrence rate compared to BCS alone [101]. Two studies reported a local recurrence rate of 40% and 33% with BCS alone without radiotherapy. The authors also suggested that cone excision alone is not a recommended procedure for MPD [111-113].

During secondary management after the lesion is resected, the decision to enhance the appearance of the breast(s) or nipple(s) through reconstruction to achieve the desired patient’s aesthetic outcomes is of critical significance. A total or skin-sparing mastectomy is often followed by breast reconstruction, such as incorporating oncoplastic surgical techniques including Grisotti mastopexy or Wise-pattern mammaplasty, integrated with a contralateral mammaplasty or mastopexy, as preferred, to preserve breast symmetry. Recently, an oncoplastic technique known as the Grisotti flap, which was found to have aesthetically satisfactory outcomes in centrally located breast cancers such as Paget’s disease, has been established. The psychological well-being of the patient is also considered when undergoing this reconstructive procedure. Procedures involving immediate or delayed nipple-areolar reconstruction (using different types of skin flaps) and/or dermatography (medical tattooing) may be performed to create a symmetrical, color-matched nipple-areolar complex following resection of the nipple and/or areola. One case report conducted a left mastectomy and SLNB with immediate ipsilateral pedicled transverse rectus abdominal muscle (TRAM) flap reconstruction. Many alternative skin flaps can be used, such as the deep inferior epigastric perforator flap (DIEP), free TRAM, and pedicle TRAM. The ideal method is the DIEP-free flap, but it has limitations because it would require the use of microvascular surgery and extensive surgeon experience [2].

Neoadjuvant chemotherapy has been recently developed, which is gaining more popularity against breast cancer and has been shown to achieve better clinical outcomes [114]. NCCN guidelines have established that chemotherapy can have a positive impact on the prognosis of MPD-IDC patients. On the contrary, chemotherapy has not been recommended for MPD-DCIS patients. Hence, underlying carcinoma is an aspect accounted for while advocating chemotherapy [115]. Most cases of MPD can be classified as HER2 overexpression subtypes. Henceforth, disease recurrence can be suppressed and survival time can be extended by utilizing hormone-sensitive chemotherapy as an adjuvant treatment that can mitigate complications by impeding tumor cells. For MPD without invasive components or with associated DCIS and ER-positive cells, low-dose tamoxifen at 5 mg once daily for three years is recommended [46]. A recent article demonstrated promising results where Glypican-3 (GPC3), a cell surface proteoglycan frequently overexpressed in certain types of cancer expression, significantly differentiated MPD and IDC from other breast cancer histological subtypes. Moreover, GPC3 expression was specifically exhibited in tumors that had positive HER2 receptors. Consequently, it has been deduced to potentially serve as a selective target for the development of novel therapeutics for subtypes of breast cancer expressing GPC3 [116].

Prognosis and complications

MPD can present complications that may significantly impact patient outcomes. The most prevalent complication is local recurrence, despite successful initial treatment. However, it is important to note that recent studies have indicated that the rate of recurrence in individuals who have undergone BCS surgery is similar to that of those who were treated with mastectomies [107]. Additionally, local recurrence rates are noted to be higher in individuals with underlying invasive carcinoma compared to those with non-invasive carcinoma [101]. This is consistent with multiple studies that point toward the data that MPD negatively affects breast cancer survival. A study conducted by Ordz-Pagan et al. emphasized this, which showed that the group with breast cancer alone had a five-year survival rate of 93.8% versus 81.2% for those with MPD [117]. Aside from local recurrence, MPD carries the risk of distant metastasis, which commonly involves bones, lungs, liver, and brain. The most common metastasis is to the lymph nodes [118]. If MPD is coupled with IDC, there is an increased risk of axillary lymph node metastasis [33]. Both local recurrence and metastasis often require aggressive modes of therapy, such as systemic chemotherapy, additional surgical procedures, and adjuvant therapies [42].

Several demographic factors were consistently associated with decreased five-year overall survival rates. These factors include race, age, and gender. People of black ethnicity had a shorter survival rate compared to their other counterparts. As the age of the afflicted individual was on the higher end of the spectrum, their survival rates also decreased [119]. While fewer males are affected by MPD, their prognosis is worse. Five-year survival rates in women are 30-50%, whereas in men they are as low as 20-30% [120]. Pathological factors that correlate with survival rates include lymph node status, tumor grade, cancer stage, and cancer metastases. Lymph node status is a significant predictor of survival rates. Patients with negative lymph nodes had a five-year survival rate of 75-95% compared to those with positive lymph nodes, with a significantly decreased percentage of 20-25% [5]. Involvement of axillary lymph nodes also points toward a poorer prognosis [33]. Individuals presenting with DCIS have a better survival rate than those with invasive carcinoma. Five-year survival rates with DCIS range from 94-98%, while those with invasive carcinoma are between 73% and 93% [121]. The correlation between these pathological factors and their effect on prognosis underscores the significance of early detection and intervention to improve patient outcomes.

## Conclusions

MPD is a rare unilateral skin lesion of the nipple-areolar complex that is associated with underlying breast carcinoma. MPD usually begins with a vague presentation like pruritus, eczema, or erythema of the nipple. As the nipple normally appears in the initial stages of the disease, it can easily be misdiagnosed as a benign dermatological condition, including dermatitis or eczema. In the later stages, erosion of the nipple-areolar complex resembles malignant skin conditions like squamous cell carcinoma and basal cell carcinoma. Usually, it is the persisting erythema and itching that raises concerns for the patients and the physicians.

MPD presents unique challenges in diagnosis and treatment that the medical faculty is forced to face. It is imperative to establish standardized diagnostic criteria to minimize misdiagnosis and avoid delayed treatment. Every patient should be provided with thorough and individualized care, considering the unique manifestation of the disease and associated risks. Future research directions should include the integration of advanced molecular diagnostics with traditional imaging and histopathology and novel therapeutic techniques.
